# Morphological engineering of self-assembled nanostructures at nanoscale on faceted GaAs nanowires by droplet epitaxy

**DOI:** 10.1186/s11671-014-0717-y

**Published:** 2015-01-27

**Authors:** Guo-Wei Zha, Li-Chun Zhang, Ying Yu, Jian-Xing Xu, Si-Hang Wei, Xiang-Jun Shang, Hai-Qiao Ni, Zhi-Chuan Niu

**Affiliations:** State Key Laboratory of Superlattices and Microstructures, Institute of Semiconductors, Chinese Academy of Sciences, P.O. Box 912, Beijing, 100083 China; Synergetic Innovation Center of Quantum Information and Quantum Physics, University of Science and Technology of China, Hefei, Anhui 230026 China

**Keywords:** Droplet epitaxy, Nanostructures, Morphology, Nanowires

## Abstract

Fabrication of advanced artificial nanomaterials is a long-term pursuit to fulfill the promises of nanomaterials and it is of utter importance to manipulate materials at nanoscale to meet urgent demands of nanostructures with designed properties. Herein, we demonstrate the morphological tailoring of self-assembled nanostructures on faceted GaAs nanowires (NWs). The NWs are deposited on different kinds of substrates. Triangular and hexagonal prism morphologies are obtained, and their corresponding {110} sidewalls act as platforms for the nucleation of gallium droplets (GDs). We demonstrate that the morphologies of the nanostructures depend not only on the annealing conditions but also on the morphologies of the NWs' sidewalls. Here, we achieve morphological engineering in the form of novel quantum dots (QDs), ‘square’ quantum rings (QRs), ‘rectangular’ QRs, 3D QRs, crescent-shaped QRs, and nano-antidots. The evolution mechanisms for the peculiar morphologies of both NWs and nanostructures are modeled and discussed in detail. This work shows the potential of combining nano-structural engineering with NWs to achieve multifunctional properties and applications.

## Background

Self-assembled III-V nanostructures have attracted extensive interest and intensive research as solid quantum light emitters owing to their stability, narrow spectral linewidth, and short radiative lifetime. Meanwhile, nanowires (NWs) are building blocks for wide applications in high-performance functional lasers [1], high-mobility field-effect transistors [2], batteries [3], photodetectors, and solar cells [4]. Much of the excitement in this area of research arises from recognition that new phenomena, multifunctionality, and unprecedented integration density are possible with nanoscale structures. Recently, many investigators have turned to the synthesis of artificial nanostructures in NW systems. With NWs, a nanostructure can be constructed by inserting a slice of lower gap semiconductor along the axial direction such as CdSe/ZnSe [5], In(Ga)As/GaAs [6], InAsP/InP [7], and GaAs/AlGaAs [8] systems or via self-assembled epitaxy on faceted NWs in the radial direction [9-11]. Further attractive strategy is tailoring the nanostructured morphologies to the formation of novel hierarchical nanostructures and, in turn, may extend potential properties and consequent applications into a new technological dimension. Among various self-assembled growth methods, droplet epitaxy [12] is acting as a flexible technique applicable to both lattice-matched and lattice-mismatched systems, yielding peculiar quantum dot (QD), quantum ring (QR), nano-antidot, concentric QR, and coupled QR topographies [13-15]. However, all the previously published work has been limited to planar membranes, and there has been rare report of integrating such interesting nanostructures in NW systems. Further investigation is needed to explore such possibility for material epitaxy and provide an advanced nanoscale platform for the device fabrication of robust quantum optoelectronic devices. Herein, we report on tailoring self-assembled nanostructured morphologies on faceted GaAs NWs. Morphological engineering include both the different NW facets and nanostructures on them. Different kinds of substrates are used, giving rise to hexagonal and triangular prism morphologies. We demonstrate that the evolution of nanostructures depends not only on the deposition conditions but also on the morphologies of the NWs' sidewalls. This work focuses on a morphology study to give insight into the underlying growth kinetics based on the proposed models instead of a quantum confinement study.

## Methods

GaAs NWs were synthesized on GaAs (001) and (111)B substrates in a solid-source MBE system employing the traditional self-catalyzed vapor-liquid-solid (VLS) [16-18] mechanism. The substrates were sputtered with a 10-nm silicon dioxide layer by magnetron or ion beam sputtering. Prior to growth, the substrates were degassed at 630°C for 10 min in order to desorb any remnant adsorbed molecules of the surface. Growth was initiated by condensation of a nominal 1 nm Ga in the pinholes of the SiO_2_ layer as catalyst, under ultralow arsenic background pressure of 2 × 10^−9^ torr. The GaAs backbones were grown at 560°C for 40 min under an As_4_/Ga flux ratio of 12.5. The deposition rate (equivalent to planar growth rates on GaAs (100) substrates) was 1.0 μm/h. Afterward, an interruption for 10 min was introduced under high-arsenic atmosphere (V/III = 18) to crystallize the gallium droplets (GDs) on the tip and induce the lateral growth of the nanowire [11]. Subsequently, the GDs were deposited again in the absence of arsenic flux and then thermally annealed for 10 min under various arsenic overpressures to form nanostructures on the sidewalls of the GaAs backbones.

## Results and discussion

**Figure 1:**
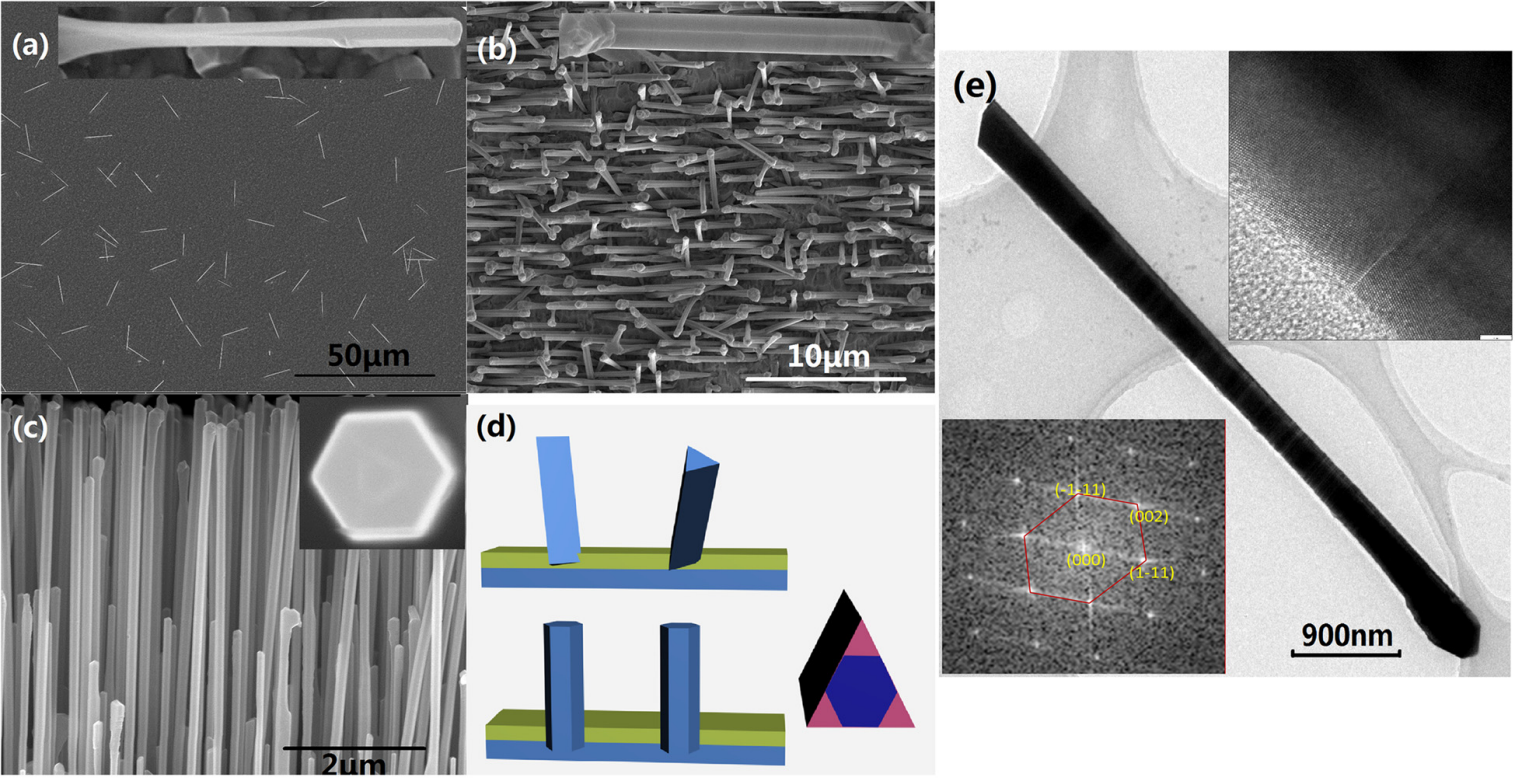
**Representative SEM images of the obtained NWs. (a)** Ion beam-sputtered (100) substrate, **(b)** magnetron-sputtered (100) substrate, and **(c)** magnetron-sputtered (111)B substrate. The inset in each panel is the magnified picture of NWs to illustrate the cross-sectional morphologies. **(d)** A schematic model for the triangular and hexagonal prism NWs on different substrates. The blue and yellow parts represent GaAs and SiO_2_, respectively. **(e)** TEM characterization of NWs in **(a)**; insets are HRTEM image and its corresponding FFTs.

Figure 1 is the representative scanning electron microscopy (SEM) images of the obtained NWs on different substrates with uniform average length of 4.5 μm and diameter of 260 nm. The difference lies in the existence or not of a preferential orientation of the NWs. Figure 1a shows that the NWs are grown in random orientations for ion beam-sputtered SiO_2_ surface. However, in the case of magnetron-sputtered samples (Figure 1b,c), most of the NWs align on the same orientations, with their growth axis following the <111> B azimuths of the underlying substrates. We suggest that the different sputtering energies adopted in these two systems are responsible for density discrepancy of SiO_2_ layer, and consequently, non-penetrating and penetrating pinholes are formed, respectively [19]. A dotted reflection high-energy electron diffraction pattern which is observed only on the magnetron-sputtered SiO_2_ layer confirmed this suggestion. Hereafter, the penetrating pinholes induce an epitaxial relationship between the obtained NWs and substrates with oriented NWs, while the non-penetrating ones, with the random-oriented NWs.

The typical NWs obtained here present a hexagonal prism morphology along the axial direction and exhibit sixfold symmetric vertical facets as verified in the insets of Figure 1b,c, commonly seen for self-catalyzed GaAs NWs [11], whereas for some other NWs, the cross section undergoes transitions from triangular to truncated triangular and then to perfect hexagonal along the axial direction (Figure 1a), all with {110} facets. Extra facets emerge and enlarge from the ridge position between adjacent threefold symmetric facets as the NWs elongate, and a sixfold symmetric geometry is formed consequently. The area per sidewall decreases by a factor of 3 in this process due to the reduction of side lengths. It is noteworthy that these two different morphologies seem to depend on the NWs' orientations: the oriented NWs are with the hexagonal morphology, whereas the random-orientated NWs, with both morphologies. Self-catalyzed GaAs NWs reported to date are hexagonal shaped [9,20,21], and this phenomenon seems to be first seen to the best of our knowledge. When growing one material epitaxially on another, the interface energies between the two materials and the surrounding medium must be considered to understand the resulting morphology [22]. We suggest that NWs preferentially grow with the morphology that minimizes their surface free energy. The triangular-shaped NWs have larger sidewalls and cross-section areas than the hexagonal NWs both with an area ratio of 9:6, based on a simplified model (Figure 1d). The effective surface free energy *Φ* is written as *Φ* = *γ*_top_ + *γ*_bottom_ + *γ*_side_. Here, *γ*_top_, *γ*_bottom_, and *γ*_side_ are the surface energy of the top GaAs layer-GDs interface, bottom GaAs layer-substrates interface, and the sidewall-vapor interface, respectively. For the oriented NWs, the penetrating pinholes and the corresponding epitaxial relationship between NWs and the underlying GaAs substrates make them a connected system. The *γ*_bottom_ should be GaAs-GaAs interface energy, and a hexagonal shape is more energetic favorable. While for the random-oriented NWs, the *γ*_bottom_ should be GaAs-SiO_2_ interface energy, which might make triangular shape a steady state or metastable state when this value increases. As the NWs elongate, the VLS growth at the NWs' tip creates a new micro-environment of the GaAs-GaAs interface, and it is reasonable that the morphological transition takes place. Rotated and truncated triangular cross sections with {112} sidewalls have been reported for Au-assisted GaAs NWs in As-rich conditions [23,24]. The sidewalls of the truncated edges are not planar surfaces but contain concave regions with {111} and {200} facets [19], which is not the case of our samples. Figure 1e shows the typical TEM image of such NWs and no concave facets are observed, although stacking faults and twin planes are found along the axis. Fast Fourier transforms (FFTs) shown in the inset confirm the {110} facets indirectly. This natural and consistent transition between different morphologies gives rise to flat planar facets, which provide a perfect platform for the nucleation of nanostructures.

**Figure 2:**
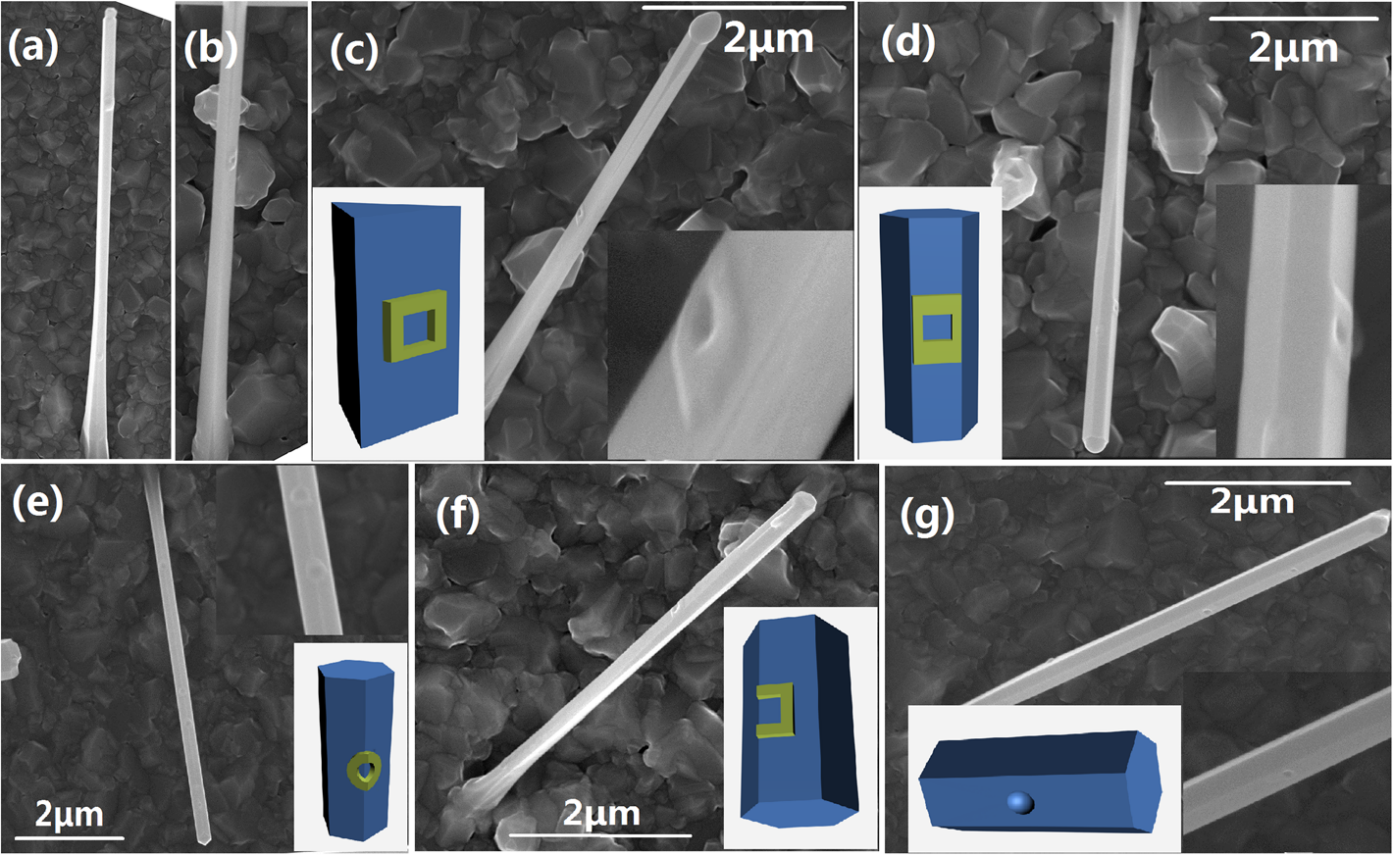
**SEM images. (a)** QD, **(b)** hole in QD, **(c)** ‘square’ QR, **(d)** ‘rectangular’ QR, **(e)** 3D QR, **(f)** crescent QR, and **(g)** nano-antidots. Insets are magnified pictures of these nanostructures or 3D schematics.

To begin, we investigate the effect of the annealing arsenic overpressure on the nanostructured morphologies. We fabricate a series of samples with varying arsenic fluxes at a fixed growth temperature of 540°C and a nominal 6-nm GD: (i) 5 × 10^−6^, (ii) 3 × 10^−6^, (iii) 2 × 10^−6^, and (iv) 9 × 10^−7^ torr. In order to obtain a more detailed picture of the surface evolution, a schematic image is plotted to show the three-dimensional (3D) features (insets) of the nanostructures for each SEM image. Figure 2a shows that QD-like islands are formed on the facets or the ridges, with base diameters (heights) of about 80 to 110 nm (10 to 25 nm). Some of them keep the original hemisphere shape of the GDs, while others show a ‘rectangular’ shape with extension along the axial direction of the NWs. Under the arsenic flux of 3 × 10^−6^ torr, a small and shallow ‘circular’ hole emerges in the midst of the former QD-like islands as shown in Figure 2b while the island around flattens and the height decreases. Lowering the arsenic flux down to 2 × 10^−6^ torr, the hole expands and deepens to form a unique QR structure. With a nominal 6-nm GD deposition, QRs on the facets of the triangular part present a perfect ‘square’ geometry (Figure 2c) with lateral lobes disconnected with the NW ridges, while a ‘rectangular’ geometry (Figure 2d) with lateral lobes connected with the NW ridges is observed for the hexagonal part. Here, both QRs have ‘rhombic’ shapes and the differences lie in the dimension of the side lengths along the axial direction of the NWs and the other orthogonal direction on the sidewalls. The central hole of the QR is a well-defined ‘circular’ or ‘square’ shape, and the QR morphology is reminiscent of the old Chinese copper coin. The outer and inner side lengths are about 90 to 120 nm and 60 to 80 nm with heights of 5 to 15 nm. For the GDs deposited on the ridges, peculiar 3D QRs are observed in Figure 2e, with a central hole deep into the ridge, while each semicircle of the QR is on the adjacent {110} sidewalls. Crescent-shaped broken QRs (Figure 2f) are formed for those GDs nucleated on the sidewalls too close to the ridges. Further lowering the arsenic flux to 9 × 10^−7^ torr, the QR lobes seem to disappear and only holed nanostructures are left as shown in Figure 2g. These nano-antidots have comparable sizes and geometry shapes to the central hole of QRs and extend about 10 nm deep into the NW backbones. Here, the nano-antidots or holed nanostructures specifically refer to nanostructures with a hole beneath the GaAs sidewalls, while QRs specifically refers to ring nanostructures with a hole above the GaAs sidewalls.

**Figure 3:**
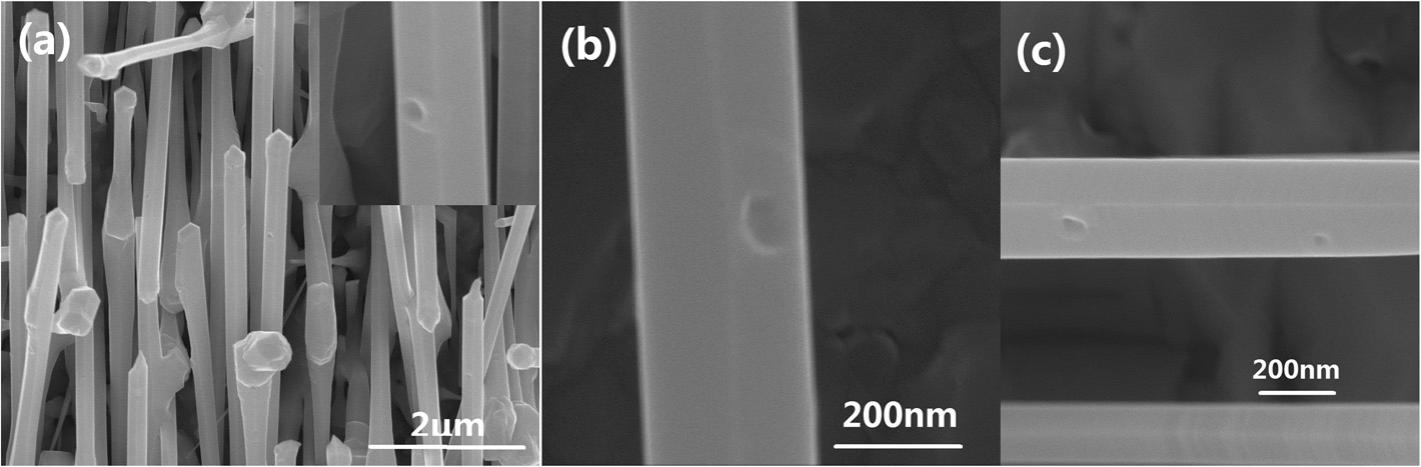
**SEM images of nanostructures on hexagonal faceted NWs. (a)** ‘Square’ QR. Nano-antidots with GD depositions of 6 nm **(b)** and 3.5 nm **(c)**.

To realize effective quantum confinement and act as practical quantum devices, the nanostructures should have well-defined size and density. Figure 3a shows an example of QR nucleated on a hexagonal facet, with a GD deposition of 3.5 nm. The QR presents a decreased outer (inner) side length of about 80 nm (45 nm), when the amount of GD deposition is decreased. Moreover, the previously mentioned extension along the axial direction is rarely seen and a more symmetric ‘square’ morphology is achieved. Figure 3b,c compares the morphology differences for nano-antidots with GD depositions of 6 and 3.5 nm, respectively. By decreasing the deposition amount, the diameter of the holes decreases and the GaAs lobes around the holes diminish and finally disappear. It might provide an advanced platform for the fabrication of patterned QDs [25] on faceted GaAs NWs and in achieving bright single-photon sources [14].

**Figure 4:**
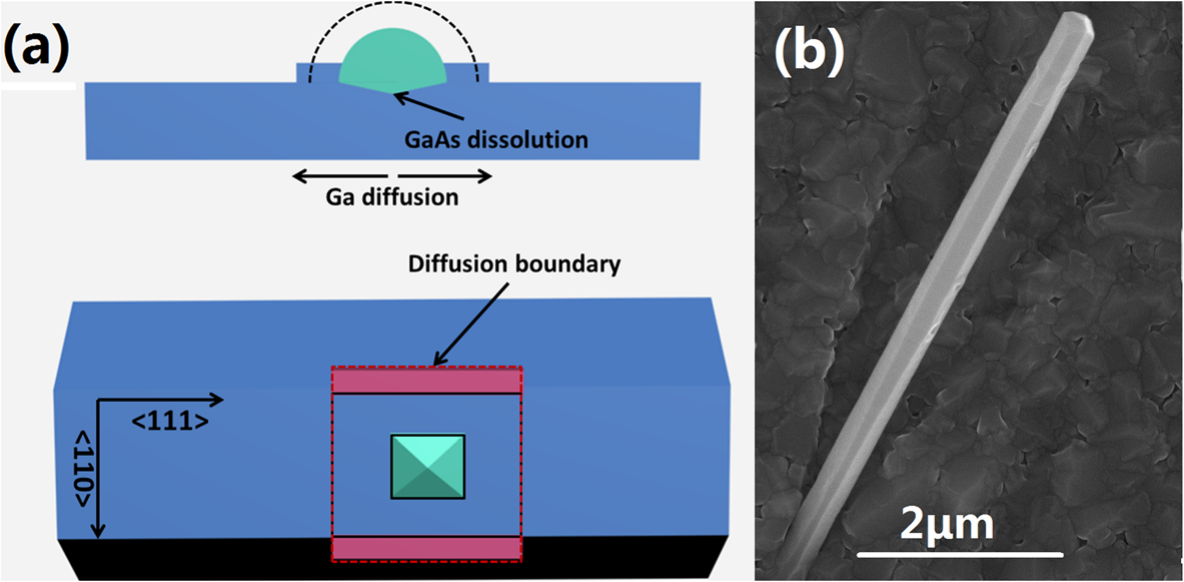
**Theoretical model and experimental verification. (a)** A schematic illustration of evolution mechanism for nanostructures on faceted GaAs NWs in both cross-sectional and front views. **(b)** SEM image of nanostructures fabricated on Al_0.7_Ga_0.3_As shell.

The evolution dependence of nanostructures on arsenic flux, sidewall morphologies, and amount of GDs casts light on the underlying growth kinetics. We suggest that the formation of nanostructures on faceted GaAs nanowires consists of two major processes: nucleation of GDs and evolution of GDs into nanostructures. As reported in our earlier studies [17], the nucleation of GDs is a strain-driven, transport-dependent process. We here emphasize on the detailed evolution mechanism of the latter process, which is particularly involved with the peculiar morphologies. It is accepted that droplet epitaxy is governed by the competition between the migration of group III adatoms and the speed of crystallization driven by the gradient of surface energy [26-29]. The interfacial tensions on the boundary of GDs make it a preferential site for gallium adatoms reacting with arsenic. Meanwhile, GaAs is unstable under Ga-rich conditions at high temperatures [30-32]. In combination with the obtained results, we proposed a modified model for the crystallization process of GDs with As ambience at high temperatures, as schematically illustrated in Figure 4a. The green, blue, and red parts represent GDs, GaAs, and the diffusion boundary, respectively. The dotted line represents the original outline of GDs before annealing. During the crystallization process, Ga adatoms diffuse along the radial direction of GDs and crystallize preferentially at the boundary of the GDs. Meanwhile, the size of the GDs shrinks, which defines the inner outline of the GaAs nanostructures. Parts of the Ga adatoms diffuse beyond the GD boundary and bond with As atoms nearby, defining the outer outline composed of the diffusion boundary. Simultaneously, the dissolution of the underneath GaAs substrates induces the collapse of GDs and defines the outline along the height direction. The dissolution rate depends on the effective amount of gallium at certain sites and thus presents an inverted-pyramid distribution along the radial direction.

In the case of nanostructures on faceted GaAs NWs, the GDs nucleate on the area-limited {110} sidewalls with <110 > and <111 > direction along the two side lengths (Figure 4a), respectively. Material epitaxy on the (110) plane differs from that on the (100) and (111)B planes [33]. First-principles calculations [34] have shown that Ga migration on GaAs (110) surface has much lower barrier energies along the <110 > directions than those along <100>. We assume that <111 > might be another preferential direction for Ga adatoms on the (110) surface. Hereafter, the diffusion zone of the Ga adatoms along the radial direction of GDs presents a ‘square’ boundary. Under sufficiently high arsenic overpressure, the diffusion is suppressed and a hemisphere QD inherits the original shape of GDs. Lowering the arsenic overpressure increases the diffusion to form a ‘square’ diffusion boundary. As previously mentioned, the hexagonal prism NWs have smaller lateral side lengths than those with a triangular prism. When a larger GD is formed and the diffusion length is beyond the lateral side lengths of the hexagonal prism NWs, the ‘square’ diffusion zone is truncated by the ridge and hereafter a ‘rectangular’ geometry of the nanostructures is achieved (Figure 4a), such as 'rectangular’ QDs and QRs. This interpretation also claims for the crescent-shaped QRs. When an ultralow arsenic overpressure is applied, the incoming flux reaches equilibrium with desorption and barely reacts with Ga adatoms. At this time, GD-assisted dissolution of the underneath GaAs facets dominates and the holed nanostructures without clear lobes are achieved consequently. As of the central holes with the geometry presenting either ‘circular’ or ‘square’, we ascribe it to their relatively smaller size, and diffusion makes this geometry difference less evident. Moreover, we expect that the proposed model might apply to heterogeneous systems. Experiments on the Al_0.7_Ga_0.3_As shell are conducted. The difference is that larger amounts of GDs (over 20 nm) and relatively lower annealing temperatures (below 500°C) are required to obtain the identical morphologies as those on GaAs facets (Figure 4b). This might be due to the larger diffusion length of Ga adatoms on {110} Al_0.7_Ga_0.3_As planes in comparison with the GaAs planes.

## Conclusions

In summary, we have investigated tailoring self-assembled nanostructured morphologies on faceted GaAs NWs. Triangular and hexagonal prism morphologies are obtained on different substrates, and their corresponding {110} sidewalls act as platforms for the nucleation of GDs. The evolution dependence of GDs during arsenic processing on arsenic flux, sidewall morphologies, amount of GDs, deposition temperature, and GaAs/AlGaAs sidewalls is demonstrated in detail. Here, we achieved morphological tailoring in the form of QDs, ‘square’ QRs, ‘rectangular’ QRs, 3D QRs, crescent-shaped QRs, and nano-antidots, most of which are reported here first. Underlying growth kinetics for the peculiar morphologies of both NWs and nanostructures are modeled and fully accounted.
